# (*meso*-5,5,7,12,12,14-Hexamethyl-1,4,8,11-tetra­azacyclo­tetra­deca­ne)nickel(II) bis­(*O*,*O*′-dibenzyl dithio­phosphate)

**DOI:** 10.1107/S1600536808008398

**Published:** 2008-04-04

**Authors:** Bin Xie, Li-Ke Zou, Yi-Guo He, Jian-Shen Feng, Xiu-Lan Zhang

**Affiliations:** a Department of Chemistry, Sichuan University of Science & Engineering, Zigong, Sichuan 643000, People’s Republic of China

## Abstract

In the title salt-type 1:2 adduct, [Ni(C_16_H_36_N_4_)](C_14_H_14_O_2_PS_2_)_2_ or [Ni(tet-a)][S_2_P(OCH_2_Ph)_2_]_2_, where tet-a is *meso*-5,5,7,12,12,14-hexa­methyl-1,4,8,11-tetra­azacyclo­tetra­decane, the [Ni(tet-a)]^2+^ complex cation exhibits a relatively undistorted square-planar geometry about the Ni atom, which lies on an inversion centre and is coordinated by four macrocyclic N atoms. The two *O*,*O*′-bis­(2-phenyl­meth­yl) dithio­phosphate anions act as counter-ions to balance the charge and they inter­act with the complex through N—H⋯S hydrogen bonds. Important geometric data include Ni—N distances of 1.958 (3) and 1.963 (3) Å.

## Related literature

For related literature, see: Burchell *et al.* (2000 ▶); Ali *et al.* (2004 ▶); Allen (2002 ▶); Li *et al.* (2006 ▶); Liu *et al.* (1997 ▶).
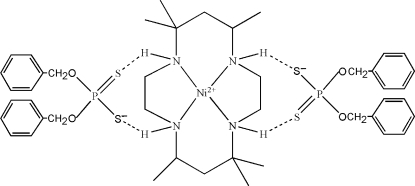


## Experimental

### 

#### Crystal data

[Ni(C_16_H_36_N_4_)](C_14_H_14_O_2_PS_2_)_2_
*M* *_r_* = 961.88
Monoclinic, 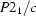

*a* = 16.371 (5) Å
*b* = 14.917 (5) Å
*c* = 9.964 (4) Å
β = 103.11 (3)°
*V* = 2369.9 (15) Å^3^
*Z* = 2
Mo *K*α radiation
μ = 0.70 mm^−1^
*T* = 290 (2) K
0.40 × 0.38 × 0.36 mm

#### Data collection

Enraf–Nonius CAD-4 diffractometer
Absorption correction: ψ scan (North *et al.*, 1968 ▶) *T*_min_ = 0.768, *T*_max_ = 0.787
5571 measured reflections
4393 independent reflections
2880 reflections with *I* > 2σ(*I*)
*R*_int_ = 0.021
3 standard reflections every 300 reflections intensity decay: 1.1%

#### Refinement

*R*[*F* ^2^ > 2σ(*F* ^2^)] = 0.049
*wR*(*F* ^2^) = 0.127
*S* = 1.01
4393 reflections
275 parameters
H-atom parameters constrained
Δρ_max_ = 0.52 e Å^−3^
Δρ_min_ = −0.34 e Å^−3^

### 

Data collection: *CAD-4 Software* (Enraf–Nonius, 1989 ▶); cell refinement: *CAD-4 Software*; data reduction: *XCAD4* (Harms & Wocadlo, 1995 ▶); program(s) used to solve structure: *SHELXS97* (Sheldrick, 2008 ▶); program(s) used to refine structure: *SHELXL97* (Sheldrick, 2008 ▶); molecular graphics: *ORTEP-3 for Windows* (Farrugia, 1997 ▶); software used to prepare material for publication: *SHELXL97*.

## Supplementary Material

Crystal structure: contains datablocks I, global. DOI: 10.1107/S1600536808008398/dn2324sup1.cif

Structure factors: contains datablocks I. DOI: 10.1107/S1600536808008398/dn2324Isup2.hkl

Additional supplementary materials:  crystallographic information; 3D view; checkCIF report

## Figures and Tables

**Table 1:** Hydrogen-bond geometry (Å, °)

*D*—H⋯*A*	*D*—H	H⋯*A*	*D*⋯*A*	*D*—H⋯*A*
N1—H1⋯S1	0.91	2.61	3.390 (3)	144
N2—H2⋯S2^i^	0.91	2.50	3.394 (3)	169

Symmetry code: (i) 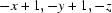.

**Fig. 1.**
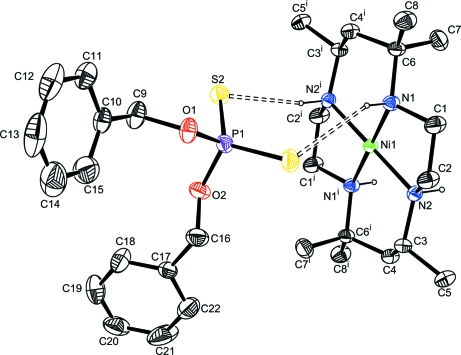
A View of the title compound showing the atom-labelling scheme.Only one of the two anions is represented for clarity.Displacement ellipsoids are drawn at the 30% probability level. Hydrogen-bonds are shown as dashed lines. For the sake of clarity, H atoms bonded to C atoms have been omitted. H atoms are represented as small spheres of arbitrary radii. [Symmetry code: (i) -x + 1, -y + 1, -z].

## supplementary crystallographic information

### Comment

Considerable interest in the chemistry of the *O*,*O'*-dialkyl dithiophosphate complexes of transition metal results from their value to industry, namely, as anti-oxidants, additives to lubricating oils, flotation reagents, insecticides(Liu *et al.*,1997;Li *et al.*,2006).We report here the synthesis and crystal structure of salt-type adduct [Ni(tet-a)][S_2_P(OCH_2_Ph)_2_]_2_ (tet-a=*meso*-5,5,7,12,12,14-hexamethyl- 1,4,8,11-tetraazacyclotetradecane, a tetradentate macrocyclic nitrogen base (Burchell *et al.*,2000; Ali *et al.*,2004 ))

The Ni^II^ in the complex cation [Ni(tet-a)]^2+^ lies on an inversion center and is coordinated by four nitrogen atoms of teteaaza macrocycle in undistorted square-planar geometry (Fig.1). The bond lengths and angles within the complex may be considered normal in comparison with the Cambridge Structural Database results (Allen, 2002).

The two *O*,*O*'- di(2-phenylmethyl) dithiophosphates act as counter-ions to balance the charge and they interact with the complex through N—H···S hydrogen bonds (Table 1).

### Experimental

A hot solution of tet-a.2H_2_O (0.77 g, 2 mmol) and Ni(OAc)_2_.2H_2_O (0.25 g, 1 mmol) in 20 ml me thanol was quickly added to a hot solution of (PhCH_2_O)_2_PSSNH_2_(C_2_H_5_)_2_(0.77 g, 2 mmol) in 20 ml me thanol. The mixture was refluxed for 6 h, then cooled to room temperature, the orange-red precipitate was collected by filtration, washed with small amounts of methanol.A solution of adduct (1) in DMSO was kept at room temperature, and red block crystals suitable for X-ray diffraction studies were obtained in eight months,.

### Refinement

All H atoms attached to C and N atom were fixed geometrically and treated as riding with C—H = 0.98 Å (methine), 0.97 Å (methylene), 0.96Å (methyl) or 0.93Å (aromatic) and N—H = 0.91 Å with *U*_iso_(H) = 1.2*U*_eq_(C, N) or *U*_iso_(H) = 1.5*U*_eq_(methyl).

### Crystal data

**Table:**

[Ni(C_16_H_36_N_4_)](C_14_H_14_O_2_PS_2_)_2_	*F*_000_ = 1020
*M**_r_* = 961.88	*D*_x_ = 1.348 Mg m^−^^3^
Monoclinic, *P*2_1_/*c*	Mo *K*α radiation λ = 0.71073 Å
Hall symbol: -P 2ybc	Cell parameters from 19 reflections
*a* = 16.371 (5) Å	θ = 4.4–5.5º
*b* = 14.917 (5) Å	µ = 0.70 mm^−^^1^
*c* = 9.964 (4) Å	*T* = 290 (2) K
β = 103.11 (3)º	Block, red
*V* = 2369.9 (15) Å^3^	0.40 × 0.38 × 0.36 mm
*Z* = 2	

### Data collection

**Table:**

Enraf–Nonius CAD-4 diffractometer	*R*_int_ = 0.021
Radiation source: fine-focus sealed tube	θ_max_ = 25.5º
Monochromator: graphite	θ_min_ = 1.3º
*T* = 290(2) K	*h* = −7→19
ω/2θ scans	*k* = −18→0
Absorption correction: ψ scan(North *et al.*, 1968)	*l* = −12→11
*T*_min_ = 0.768, *T*_max_ = 0.787	3 standard reflections
5571 measured reflections	every 300 reflections
4393 independent reflections	intensity decay: 1.1%
2880 reflections with *I* > 2σ(*I*)	

### Refinement

**Table:**

Refinement on *F*^2^	Secondary atom site location: difference Fourier map
Least-squares matrix: full	Hydrogen site location: inferred from neighbouring sites
*R*[*F*^2^ > 2σ(*F*^2^)] = 0.049	H-atom parameters constrained
*wR*(*F*^2^) = 0.127	*w* = 1/[σ^2^(*F*_o_^2^) + (0.0689*P*)^2^] where *P* = (*F*_o_^2^ + 2*F*_c_^2^)/3
*S* = 1.01	(Δ/σ)_max_ < 0.001
4393 reflections	Δρ_max_ = 0.52 e Å^−^^3^
275 parameters	Δρ_min_ = −0.34 e Å^−^^3^
Primary atom site location: structure-invariant direct methods	Extinction correction: none

### Special details

**Table:**

Geometry. All e.s.d.'s (except the e.s.d. in the dihedral angle between two l.s. planes) are estimated using the full covariance matrix. The cell e.s.d.'s are taken into account individually in the estimation of e.s.d.'s in distances, angles and torsion angles; correlations between e.s.d.'s in cell parameters are only used when they are defined by crystal symmetry. An approximate (isotropic) treatment of cell e.s.d.'s is used for estimating e.s.d.'s involving l.s. planes.
Refinement. Refinement of *F*^2^ against ALL reflections. The weighted *R*-factor *wR* and goodness of fit *S* are based on *F*^2^, conventional *R*-factors *R* are based on *F*, with *F* set to zero for negative *F*^2^. The threshold expression of *F*^2^ > σ(*F*^2^) is used only for calculating *R*-factors(gt) *etc*. and is not relevant to the choice of reflections for refinement. *R*-factors based on *F*^2^ are statistically about twice as large as those based on *F*, and *R*- factors based on ALL data will be even larger.

### Fractional atomic coordinates and isotropic or equivalent isotropic displacement parameters (Å^2^)

**Table:**

	*x*	*y*	*z*	*U*_iso_*/*U*_eq_	
Ni1	0.5000	0.5000	0.0000	0.02975 (18)	
S1	0.39590 (6)	0.45749 (7)	0.26928 (12)	0.0569 (3)	
S2	0.28652 (6)	0.64383 (7)	0.13356 (11)	0.0498 (3)	
P1	0.29136 (6)	0.52753 (7)	0.22790 (10)	0.0406 (3)	
O1	0.26427 (15)	0.53665 (18)	0.3726 (3)	0.0509 (7)	
O2	0.21601 (15)	0.47114 (17)	0.1325 (3)	0.0521 (7)	
N1	0.57052 (16)	0.53521 (17)	0.1790 (3)	0.0341 (7)	
H1	0.5357	0.5294	0.2379	0.041*	
N2	0.55476 (16)	0.38269 (17)	0.0308 (3)	0.0332 (6)	
H2	0.5975	0.3845	−0.0138	0.040*	
C1	0.6346 (2)	0.4648 (2)	0.2251 (4)	0.0543 (12)	
H1A	0.6539	0.4663	0.3245	0.065*	
H1B	0.6824	0.4747	0.1844	0.065*	
C2	0.5955 (2)	0.3765 (2)	0.1806 (4)	0.0478 (10)	
H2A	0.5540	0.3619	0.2330	0.057*	
H2B	0.6378	0.3298	0.1960	0.057*	
C3	0.5074 (2)	0.2981 (2)	−0.0165 (3)	0.0353 (8)	
H3	0.4636	0.2913	0.0354	0.042*	
C4	0.4652 (2)	0.3050 (2)	−0.1675 (4)	0.0397 (9)	
H4A	0.4439	0.2462	−0.1989	0.048*	
H4B	0.5077	0.3204	−0.2174	0.048*	
C5	0.5636 (2)	0.2146 (2)	0.0096 (4)	0.0452 (9)	
H5A	0.6067	0.2194	−0.0413	0.068*	
H5B	0.5304	0.1621	−0.0197	0.068*	
H5C	0.5889	0.2101	0.1062	0.068*	
C6	0.6063 (2)	0.6282 (2)	0.2081 (4)	0.0359 (8)	
C7	0.6734 (2)	0.6441 (3)	0.1263 (4)	0.0542 (11)	
H7A	0.6523	0.6262	0.0323	0.081*	
H7B	0.7225	0.6096	0.1658	0.081*	
H7C	0.6877	0.7066	0.1293	0.081*	
C8	0.6443 (2)	0.6385 (3)	0.3636 (4)	0.0543 (11)	
H8A	0.6602	0.6998	0.3838	0.081*	
H8B	0.6928	0.6008	0.3896	0.081*	
H8C	0.6035	0.6212	0.4144	0.081*	
C9	0.1889 (3)	0.5872 (3)	0.3782 (4)	0.0624 (12)	
H9A	0.1446	0.5736	0.2982	0.075*	
H9B	0.2003	0.6510	0.3783	0.075*	
C10	0.1620 (2)	0.5621 (3)	0.5064 (4)	0.0511 (10)	
C11	0.1766 (3)	0.6179 (3)	0.6203 (5)	0.0639 (12)	
H11	0.2020	0.6734	0.6175	0.077*	
C12	0.1527 (3)	0.5897 (5)	0.7399 (5)	0.0884 (18)	
H12	0.1623	0.6266	0.8171	0.106*	
C13	0.1153 (4)	0.5082 (6)	0.7433 (7)	0.103 (2)	
H13	0.0995	0.4899	0.8230	0.123*	
C14	0.1010 (4)	0.4537 (5)	0.6318 (8)	0.108 (2)	
H14	0.0759	0.3980	0.6349	0.130*	
C15	0.1237 (3)	0.4810 (4)	0.5149 (6)	0.0822 (15)	
H15	0.1130	0.4435	0.4383	0.099*	
C16	0.2008 (2)	0.3800 (3)	0.1649 (5)	0.0604 (12)	
H16A	0.1953	0.3756	0.2596	0.072*	
H16B	0.2473	0.3425	0.1542	0.072*	
C17	0.1214 (2)	0.3495 (2)	0.0688 (4)	0.0472 (10)	
C18	0.0461 (3)	0.3895 (3)	0.0682 (5)	0.0681 (13)	
H18	0.0439	0.4365	0.1287	0.082*	
C19	−0.0272 (3)	0.3613 (4)	−0.0213 (5)	0.0795 (16)	
H19	−0.0781	0.3884	−0.0193	0.095*	
C20	−0.0245 (3)	0.2952 (4)	−0.1100 (6)	0.0853 (18)	
H20	−0.0738	0.2751	−0.1682	0.102*	
C21	0.0503 (4)	0.2567 (4)	−0.1159 (6)	0.108 (2)	
H21	0.0522	0.2126	−0.1811	0.130*	
C22	0.1229 (3)	0.2831 (3)	−0.0256 (6)	0.0808 (16)	
H22	0.1734	0.2557	−0.0286	0.097*	

### Atomic displacement parameters (Å^2^)

**Table:**

	*U*^11^	*U*^22^	*U*^33^	*U*^12^	*U*^13^	*U*^23^
Ni1	0.0297 (3)	0.0263 (3)	0.0308 (3)	0.0014 (2)	0.0017 (2)	0.0013 (3)
S1	0.0404 (5)	0.0557 (6)	0.0758 (8)	0.0085 (5)	0.0157 (5)	0.0121 (6)
S2	0.0506 (6)	0.0459 (6)	0.0535 (6)	−0.0007 (4)	0.0129 (5)	0.0074 (5)
P1	0.0349 (5)	0.0421 (5)	0.0440 (6)	−0.0003 (4)	0.0070 (4)	0.0019 (4)
O1	0.0459 (14)	0.0648 (17)	0.0443 (16)	0.0157 (13)	0.0152 (12)	0.0131 (13)
O2	0.0455 (14)	0.0413 (14)	0.0633 (18)	−0.0079 (11)	−0.0011 (13)	0.0018 (13)
N1	0.0367 (14)	0.0292 (14)	0.0336 (16)	0.0000 (12)	0.0023 (12)	0.0011 (12)
N2	0.0332 (14)	0.0297 (15)	0.0358 (16)	0.0036 (11)	0.0059 (12)	0.0022 (12)
C1	0.052 (2)	0.037 (2)	0.059 (3)	0.0101 (17)	−0.019 (2)	−0.0037 (19)
C2	0.058 (2)	0.035 (2)	0.039 (2)	0.0084 (17)	−0.0113 (18)	0.0016 (17)
C3	0.0396 (18)	0.0274 (16)	0.038 (2)	−0.0049 (14)	0.0082 (15)	−0.0018 (15)
C4	0.0458 (19)	0.0307 (18)	0.041 (2)	−0.0024 (15)	0.0071 (16)	−0.0022 (16)
C5	0.060 (2)	0.0301 (19)	0.044 (2)	0.0053 (16)	0.0098 (18)	0.0000 (17)
C6	0.0390 (18)	0.0301 (18)	0.037 (2)	−0.0041 (14)	0.0046 (15)	−0.0051 (15)
C7	0.041 (2)	0.056 (2)	0.065 (3)	−0.0041 (18)	0.0109 (19)	0.006 (2)
C8	0.058 (2)	0.050 (2)	0.045 (2)	0.0027 (19)	−0.0075 (19)	−0.0090 (19)
C9	0.060 (2)	0.075 (3)	0.056 (3)	0.025 (2)	0.020 (2)	0.014 (2)
C10	0.044 (2)	0.061 (3)	0.050 (3)	0.019 (2)	0.0147 (18)	0.011 (2)
C11	0.058 (3)	0.073 (3)	0.060 (3)	0.019 (2)	0.012 (2)	−0.001 (2)
C12	0.083 (4)	0.128 (5)	0.055 (3)	0.050 (4)	0.018 (3)	−0.003 (3)
C13	0.097 (4)	0.138 (6)	0.091 (5)	0.063 (4)	0.061 (4)	0.053 (5)
C14	0.114 (5)	0.089 (5)	0.146 (6)	0.021 (4)	0.080 (5)	0.043 (5)
C15	0.095 (4)	0.077 (4)	0.086 (4)	−0.002 (3)	0.043 (3)	0.001 (3)
C16	0.055 (2)	0.039 (2)	0.081 (3)	−0.0046 (18)	0.004 (2)	0.005 (2)
C17	0.043 (2)	0.038 (2)	0.059 (3)	−0.0055 (17)	0.0095 (18)	0.0008 (19)
C18	0.060 (3)	0.079 (3)	0.064 (3)	0.020 (2)	0.010 (2)	−0.016 (3)
C19	0.043 (2)	0.118 (5)	0.076 (4)	0.011 (3)	0.009 (2)	0.010 (3)
C20	0.066 (3)	0.060 (3)	0.114 (5)	−0.018 (3)	−0.014 (3)	0.002 (3)
C21	0.113 (5)	0.062 (3)	0.126 (5)	0.007 (3)	−0.023 (4)	−0.044 (4)
C22	0.064 (3)	0.062 (3)	0.109 (4)	0.012 (2)	0.006 (3)	−0.021 (3)

### Geometric parameters (Å, °)

**Table:**

Ni1—N2	1.958 (3)	C7—H7C	0.9600
Ni1—N1	1.963 (3)	C8—H8A	0.9600
S1—P1	1.9674 (14)	C8—H8B	0.9600
S2—P1	1.9661 (15)	C8—H8C	0.9600
P1—O1	1.608 (3)	C9—C10	1.490 (6)
P1—O2	1.613 (3)	C9—H9A	0.9700
O1—C9	1.459 (4)	C9—H9B	0.9700
O2—C16	1.432 (4)	C10—C15	1.374 (7)
N1—C1	1.482 (4)	C10—C11	1.385 (6)
N1—C6	1.509 (4)	C11—C12	1.400 (7)
N1—H1	0.9100	C11—H11	0.9300
N2—C2	1.493 (4)	C12—C13	1.366 (9)
N2—C3	1.501 (4)	C12—H12	0.9300
N2—H2	0.9100	C13—C14	1.354 (9)
C1—C2	1.488 (5)	C13—H13	0.9300
C1—H1A	0.9700	C14—C15	1.363 (8)
C1—H1B	0.9700	C14—H14	0.9300
C2—H2A	0.9700	C15—H15	0.9300
C2—H2B	0.9700	C16—C17	1.500 (5)
C3—C4	1.510 (4)	C16—H16A	0.9700
C3—C5	1.535 (4)	C16—H16B	0.9700
C3—H3	0.9800	C17—C18	1.369 (5)
C4—C6^i^	1.520 (4)	C17—C22	1.370 (6)
C4—H4A	0.9700	C18—C19	1.388 (6)
C4—H4B	0.9700	C18—H18	0.9300
C5—H5A	0.9600	C19—C20	1.332 (7)
C5—H5B	0.9600	C19—H19	0.9300
C5—H5C	0.9600	C20—C21	1.366 (7)
C6—C4^i^	1.520 (4)	C20—H20	0.9300
C6—C7	1.528 (5)	C21—C22	1.376 (6)
C6—C8	1.541 (5)	C21—H21	0.9300
C7—H7A	0.9600	C22—H22	0.9300
C7—H7B	0.9600		
			
N2—Ni1—N1	86.71 (11)	C6—C7—H7B	109.5
N2^i^—Ni1—N1	93.29 (11)	H7A—C7—H7B	109.5
O1—P1—O2	104.07 (15)	C6—C7—H7C	109.5
O1—P1—S2	111.40 (12)	H7A—C7—H7C	109.5
O2—P1—S2	103.65 (11)	H7B—C7—H7C	109.5
O1—P1—S1	105.11 (11)	C6—C8—H8A	109.5
O2—P1—S1	111.05 (11)	C6—C8—H8B	109.5
S2—P1—S1	120.50 (7)	H8A—C8—H8B	109.5
C9—O1—P1	119.1 (2)	C6—C8—H8C	109.5
C16—O2—P1	120.7 (2)	H8A—C8—H8C	109.5
C1—N1—C6	112.0 (2)	H8B—C8—H8C	109.5
C1—N1—Ni1	108.7 (2)	O1—C9—C10	108.6 (3)
C6—N1—Ni1	122.9 (2)	O1—C9—H9A	110.0
C1—N1—H1	103.6	C10—C9—H9A	110.0
C6—N1—H1	103.6	O1—C9—H9B	110.0
Ni1—N1—H1	103.6	C10—C9—H9B	110.0
C2—N2—C3	110.1 (2)	H9A—C9—H9B	108.3
C2—N2—Ni1	107.3 (2)	C15—C10—C11	118.4 (4)
C3—N2—Ni1	121.11 (19)	C15—C10—C9	120.1 (4)
C2—N2—H2	105.8	C11—C10—C9	121.5 (4)
C3—N2—H2	105.8	C10—C11—C12	119.2 (5)
Ni1—N2—H2	105.8	C10—C11—H11	120.4
N1—C1—C2	108.0 (3)	C12—C11—H11	120.4
N1—C1—H1A	110.1	C13—C12—C11	120.1 (6)
C2—C1—H1A	110.1	C13—C12—H12	119.9
N1—C1—H1B	110.1	C11—C12—H12	119.9
C2—C1—H1B	110.1	C14—C13—C12	120.6 (6)
H1A—C1—H1B	108.4	C14—C13—H13	119.7
C1—C2—N2	107.9 (3)	C12—C13—H13	119.7
C1—C2—H2A	110.1	C13—C14—C15	119.5 (6)
N2—C2—H2A	110.1	C13—C14—H14	120.2
C1—C2—H2B	110.1	C15—C14—H14	120.2
N2—C2—H2B	110.1	C14—C15—C10	122.2 (6)
H2A—C2—H2B	108.4	C14—C15—H15	118.9
N2—C3—C4	110.0 (3)	C10—C15—H15	118.9
N2—C3—C5	112.4 (2)	O2—C16—C17	108.3 (3)
C4—C3—C5	110.3 (3)	O2—C16—H16A	110.0
N2—C3—H3	108.0	C17—C16—H16A	110.0
C4—C3—H3	108.0	O2—C16—H16B	110.0
C5—C3—H3	108.0	C17—C16—H16B	110.0
C3—C4—C6^i^	117.4 (3)	H16A—C16—H16B	108.4
C3—C4—H4A	107.9	C18—C17—C22	118.0 (4)
C6^i^—C4—H4A	107.9	C18—C17—C16	121.3 (4)
C3—C4—H4B	107.9	C22—C17—C16	120.6 (4)
C6^i^—C4—H4B	107.9	C17—C18—C19	121.1 (4)
H4A—C4—H4B	107.2	C17—C18—H18	119.4
C3—C5—H5A	109.5	C19—C18—H18	119.4
C3—C5—H5B	109.5	C20—C19—C18	119.8 (5)
H5A—C5—H5B	109.5	C20—C19—H19	120.1
C3—C5—H5C	109.5	C18—C19—H19	120.1
H5A—C5—H5C	109.5	C19—C20—C21	120.3 (5)
H5B—C5—H5C	109.5	C19—C20—H20	119.8
N1—C6—C4^i^	108.0 (2)	C21—C20—H20	119.8
N1—C6—C7	109.6 (3)	C20—C21—C22	120.1 (5)
C4^i^—C6—C7	111.1 (3)	C20—C21—H21	120.0
N1—C6—C8	109.6 (3)	C22—C21—H21	120.0
C4^i^—C6—C8	108.3 (3)	C17—C22—C21	120.6 (5)
C7—C6—C8	110.3 (3)	C17—C22—H22	119.7
C6—C7—H7A	109.5	C21—C22—H22	119.7

Symmetry codes: (i) −*x*+1, −*y*+1, −*z*.

### Hydrogen-bond geometry (Å, °)

**Table:**

*D*—H···*A*	*D*—H	H···*A*	*D*···*A*	*D*—H···*A*
N1—H1···S1	0.91	2.61	3.390 (3)	144
N2—H2···S2^i^	0.91	2.50	3.394 (3)	169

Symmetry codes: (i) −*x*+1, −*y*+1, −*z*.
